# Gurjun Balsam in Gonorrhœa

**Published:** 1878-03-20

**Authors:** 


### GURJUN BALSAM IN GONORRHŒA.

M. Vidal (*Journal de Medecine, December*, 1877) is the first in France who has studied the applications of this new remedy, whose remarkable properties will certainly bring it into use speedily. It is obtained from several resinous trees in the Indian Archipelago, is very abundant, and the price is moderate.

Gurjun balsam has been successfully employed for leprosy by several English physicians in India, and Vidal has also had good results from its use in the *Hopital Saint Louis*. But it is especially in gonorrhœa that it renders the greatest service. M. Deval, a student of Vidal, gives ten cases as proof of its value, and his testimony is corroborated by Maurice and others. The duration of treatment varied from ten to twenty days, the duration being shorter in proportion as the patient had passed the inflammatory stage. Vidal's formula is:

R.—Gurjun balsam (wood oil).
Acacia.................... aa. 4 grammes.
Infusion of anise seed..........40 grammes.

To be taken before meals. It was not necessary to increase the dose, which is perfectly well tolerated, the only effect being to cause one or two stools two hours after the meal. When the dose was increased, no more than eight grammes were given. Sometimes, at first a little nausea was produced, but this speedily disappeared. Vidal gives a little wine after the potion, which makes it better tolerated. No change in diet is necessary. Besides the potion, a liniment of equal parts of the balsam and lime water, applied by means of tampons, was used in women with vaginitis; the tampons were left in the vagina twenty-four hours. A cure was always rapid in women. Its advantages over copaiba are its more rapid and certain action; it does not produce erythema, and it does not give to the breath the tell-tale odor of copaiba. Its local action in vaginitis and balanitis is also excellent.—*Chicago Med. Ex.*

